# Computer-Aided Assessment of Tumor Grade for Breast Cancer in Ultrasound Images

**DOI:** 10.1155/2015/914091

**Published:** 2015-02-24

**Authors:** Dar-Ren Chen, Cheng-Liang Chien, Yan-Fu Kuo

**Affiliations:** ^1^Comprehensive Breast Cancer Center, Department of Medical Research, Changhua Christian Hospital, 135 Nanhsiao Street, Changhua 50006, Taiwan; ^2^Department of Bio-Industrial Mechatronics Engineering, National Taiwan University, No. 1, Sec. 4, Roosevelt Road, Taipei 106, Taiwan

## Abstract

This study involved developing a computer-aided diagnosis (CAD) system for discriminating the grades of breast cancer tumors in ultrasound (US) images. Histological tumor grades of breast cancer lesions are standard prognostic indicators. Tumor grade information enables physicians to determine appropriate treatments for their patients. US imaging is a noninvasive approach to breast cancer examination. In this study, 148 3-dimensional US images of malignant breast tumors were obtained. Textural, morphological, ellipsoid fitting, and posterior acoustic features were quantified to characterize the tumor masses. A support vector machine was developed to classify breast tumor grades as either low or high. The proposed CAD system achieved an accuracy of 85.14% (126/148), a sensitivity of 79.31% (23/29), a specificity of 86.55% (103/119), and an *A*_*Z*_ of 0.7940.

## 1. Introduction

Breast cancer is a leading cause of death in women worldwide [1]. The histological grade of a breast cancer tumor is regarded as a crucial prognostic indicator [2]. Rapid and accurate assessment of tumor grades is crucial for enabling a physician to determine the appropriate treatment options for patients. Previous studies have reported ultrasound (US) imaging to be an effective supplement to mammography in screening for breast cancer [3–5]. This study involved developing a computer-aided diagnosis (CAD) [6] system for assessing the tumor grades of breast cancer according to US images.

A histological tumor grade is a measure of the differentiation between cancerous and normal cells [2]. The Nottingham system [7] categorizes breast cancer into 3 grades. In general, cancer of lower grades tends to be less aggressive than cancer of higher grades. The grade of a tumor is typically determined through a morphological assessment of biopsied tissue and cells performed by pathologists using a microscope. The grading process is invasive and time consuming and can be subjective. Assessing the grades of breast cancer tumors online by using noninvasive approaches is more desirable.

Research has indicated that tumor grades are correlated with sonographic characteristics. Lamb et al. [8] observed that high-grade tumors exhibited posterior enhancement and well-defined margins. Kim et al. [9] demonstrated that parallel orientation and echo patterns were correlated with tumor grades and certain biological markers in breast cancer. Aho et al. [10] indicated that infiltrating ductal carcinoma tumors that exhibited posterior shadowing in US images were likely to be low-grade ones. Wojcinski et al. [11] evaluated the interrelationship between tumor grades and BI-RADS [12] features and determined that high-grade tumors were associated with strong posterior acoustic enhancement and weak shadowing. Chang et al. [13] quantified stellate features by using US images and observed that masses of breast cancer associated with stellate features tended to be low-grade tumors. Another study revealed that the presence of posterior enhancement in US images was correlated with an increased likelihood of the tumor being of a high grade [14].

In this study, a CAD system was developed to determine the tumor grades of the breast cancer masses captured in 3-dimensional (3D) US images. The specific objectives were to (1) quantify features of breast cancer lesions in US images, (2) identify a set of US image features that significantly correlate with tumor grades, and (3) develop a model that can be applied to distinguish between high-grade and low-grade tumors. In this study, volumetric US breast images were collected. The tumor lesions were segmented, and the features of these tumor masses were quantified. A support vector machine (SVM) classifier was developed to distinguish tumor grades, and a genetic algorithm (GA) was used for feature selection and model parameter optimization.

## 2. Materials and Methods

### 2.1. Volumetric Ultrasound Image Acquisition

The breast US images used in this study were samples of diagnostic cases obtained during routine clinical care at Changhua Christian Hospital (Changhua, Taiwan). A total of 148 cases were examined. The images were acquired using a US scanner (Voluson 730; GE Healthcare, Zipf, Austria) equipped with a 5.6–18 MHz volume transducer (RSP6-16; GE Healthcare, Zipf, Austria). The images were quantized into 256 gray levels, and the mean voxel resolution was 0.2 mm on each side. Regarding patients that exhibited multiple tumor masses, only images of the largest lesions were included in the study. The lesion sizes ranged from 0.134 to 24.061 cm^3^ (median: 2.669 cm^3^). The grades of the tumors were identified based on pathological diagnoses, which involved biopsy methods and the Nottingham grading system. The numbers of grade I, II, and III tumors were 25, 94, and 29, respectively. In this study, grades I and II were defined as low-grade, whereas grade III was considered as high grade. The images were collected between June 2007 and August 2009. The ages of the patients ranged from 24 to 87 years (median: 49 years). The ethics committee of the hospital approved the study. No patient identifications were disclosed to avoid diagnosis bias and ensure patient privacy.

### 2.2. Tumor Segmentation

**Figure 1:**
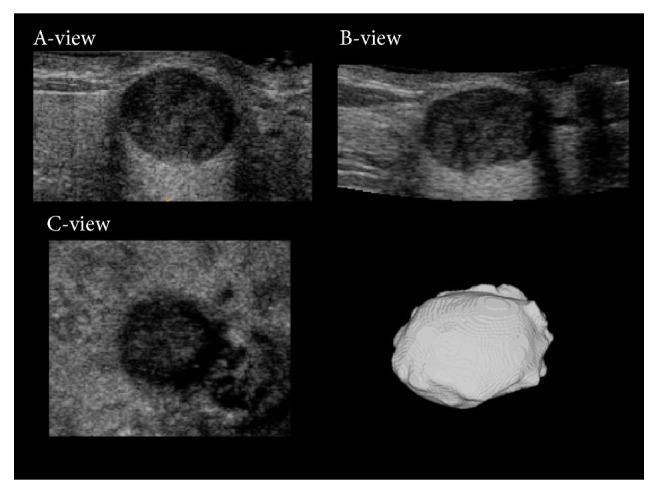
The sonographic A-view, B-view, and C-view images and a segmented volumetric tumor mass.

Segmentation was performed to extract the tumor lesions in the US images. The tumor masses were segmented semiautomatically by using ITK-SNAP [15], which performed active contouring based on a level set algorithm [16–19]. During the segmentation process, the operators identified the lesions in the US images and placed seeds (i.e., starting points) at appropriate locations inside the tumor masses. The seeds expanded until they reached the tumor boundaries. Appropriate control parameters were set to ensure that optimal segmentation results were attained [15]. Compared with manual methods, semiautomatic segmentation is more consistent and less laborious when accurate contours must be sketched. Semiautomatic segmentation is particularly suitable for use with 3D US images.  Figure 1 shows a segmented volumetric tumor mass. Experienced radiologists verified the segmentation results.

### 2.3. Feature Quantification

Features were quantified to describe the characteristics of the tumors. The features were categorized into 4 types: textural, morphological, ellipsoid fitting, and posterior acoustic. The textural features represent the spatial correlations in gray level among the voxels of a tumor mass. The textural features were calculated using a gray level cooccurrence matrix (GLCM) [20]. During this process, the gray level of the US image subjected to analysis was reduced from 256 to 16. The frequencies of the gray level differences between 2 adjacent voxels in the image were then cumulated to form the GLCM **P**_*d*_ ∈ ℜ^16×16^, where *d* ∈ ℜ^3^ is the displacement vector that represents the geometric relationship between the 2 adjacent voxels [21]. Six textural features, namely, the angular second moment *T*_ASM_, contrast *T*_Con_, inverse difference moment *T*_*I*_, entropy *T*_*E*_, dissimilarity *T*_*D*_, and correlation *T*_Cor_ [20, 22], were then calculated using **P**_*d*_. In this study, 4 displacement vectors *d* were considered: (1,1, 1), (1,0, 0), (0,1, 0), and (0,0, 1). Thus, 24 textural features were quantified.

Morphological features [23, 24] describe the superficial regularity of the tumor masses. Six morphological features were included in this study. Volume *M*_*V*_ (unit: mm^3^) and surface area *M*_*A*_ (unit: mm^2^) described the basic structural characteristics of a tumor mass. Classical compactness *M*_Cc_ was used to measure the degree of similarity between a tumor mass and its optimally fitted sphere, whereas discrete compactness *M*_Cd_ was used to evaluate the degree of similarity between a tumor mass and its optimally fitted cube [24, 25]. The mean radius *M*_*Rm*_ and standard deviation of radius *M*_*R*std_ characterized the size and surface irregularity of tumor masses.

**Figure 2:**
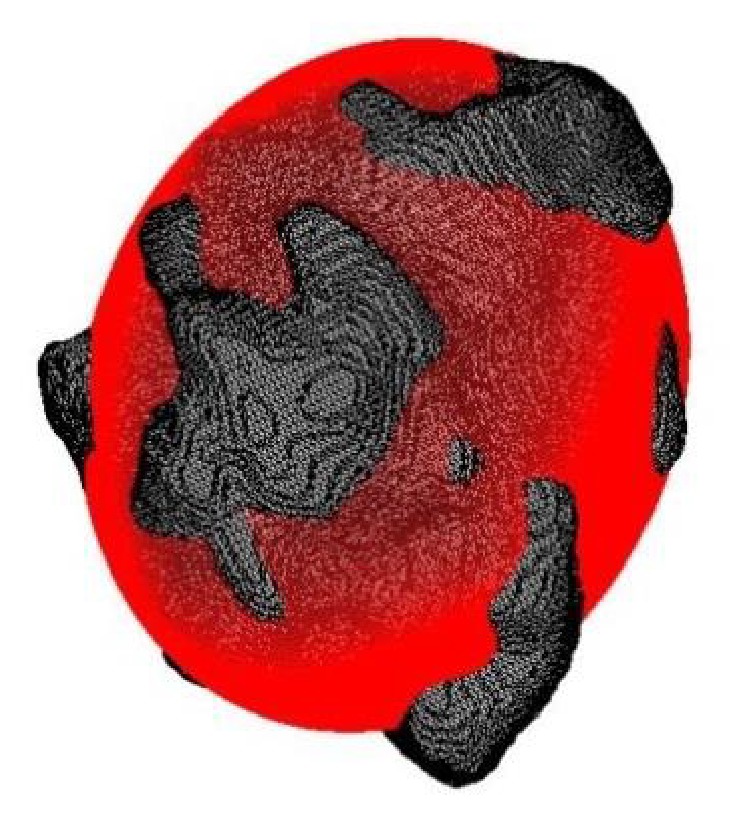
A tumor mass (gray) and its optimally fitted ellipsoid (red).

Ellipsoid fitting features [24] depict the degree of similarity between a tumor mass and its optimally fitted ellipsoid (Figure 2). The optimally fitted ellipsoid can be regarded as the baseline against which the degree of shape irregularity of a tumor mass can be measured. Nine ellipsoid fitting features were quantified: axis ratio *E*_*A*_, surface ratio *E*_*S*_, volume covering ratio *E*_*V*_, number of regions outside the ellipsoid *E*_*RO*_, number of regions inside the ellipsoid *E*_*RI*_, number of total regions *E*_*R*_, number of regions with angularity outside the ellipsoid *E*_*ROa*_, number of regions with angularity inside the ellipsoid *E*_*RIa*_, and number of total regions with angularity *E*_*Ra*_. The parameter *E*_*V*_ was defined as the ratio of the volume of the intersection between the tumor and the ellipsoid volume to the tumor volume; *E*_*R*_ is the sum of *E*_*RO*_ and *E*_*RI*_; and *E*_*Ra*_ is the sum of *E*_*ROa*_ and *E*_*RIa*_.

**Figure 3:**
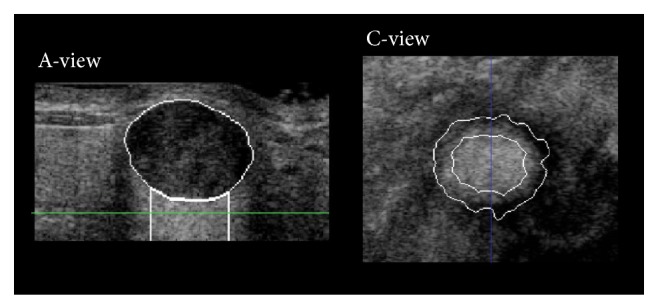
Sonographic A-view and C-view images of a tumor mass and its posterior region. The posterior region is the area under the tumor in the A-view image. The C-view image shows the section contour of the tumor lesion (external curve) and the section contour of the posterior regions (internal curve). The blue line in the C-view image indicates the plane of the A-view image. The green line in the A-view image indicates the plane of the C-view image.

Posterior acoustic features [26–28] are characterized by the discrepancy in the gray levels of a voxel between a tumor mass and its corresponding posterior region (the region beneath the tumor in the A-view image in Figure 3). When acoustic enhancement occurs, the gray level of the posterior region is greater than the gray level of the lesion in ultrasound images [29]. Five posterior acoustic features were defined: the standard deviation of the gray levels in the posterior region *P*_std_, the ratio of the mean gray level in the posterior region to that in the tumor region *P*_*Rm*_, the ratio of the gray level standard deviation in the posterior region to that in the tumor region *P*_*R*std_, the difference between the gray level means of the posterior and tumor regions *P*_*Sm*_, and the difference between the gray level standard deviations of the posterior and tumor regions *P*_*S*std_. In this study, the section area (C-view image in Figure 3) of the posterior region was defined as two-thirds of the maximum tumor mass section area to avoid the edge-shadowing effect [26, 28]. The section area of the posterior region was derived using distance transform [30]. The height of the posterior region was defined as the tumor mass height and could not exceed 100 voxels [28].

### 2.4. Tumor Grade Classification and Attribute Selection

Soft-margin SVM classifiers with radial basis function kernels were developed to differentiate between high-grade and low-grade tumors. Because the dataset used in this study was unbalanced (119 low-grade tumors and 29 high-grade tumors), the soft-margin parameter ratio was set as the reciprocal of the tumor number ratio between the 2 grades [31]. The classifiers were developed using LIBSVM [32]. During the model development process, a GA was applied to identify an optimal set of features as the model inputs and to determine the soft-margin and kernel parameters for the model [33]. Feature selection is crucial for the performance of CAD systems. Including inappropriate attributes can result in an overfitted model [34] and can therefore reduce the system performance. In this study, the fitness function [33] of the GA was set as a linear combination of the product of the model sensitivity and specificity (with a weight of 0.8) and the reciprocal of the number of selected features (with a weight of 0.2). The calculation was performed using MATLAB (MathWorks, Inc.).

### 2.5. Performance Assessment

Receiver operating characteristic analysis was applied to measure the performance levels of the CAD systems by using tenfold cross validation (CV). Six indices were calculated: the area under the curve (*A*_*Z*_), accuracy, sensitivity, specificity, positive predictive value (PPV), and negative predictive value (NPV) [35–37]. The sensitivity and the specificity were defined as the percentages of actual high-grade and low-grade tumors, respectively, that were correctly classified. The PPV and the NPV were defined as the percentages of predicted high-grade and low-grade tumors, respectively, that were correctly classified.

## 3. Results and Discussion

### 3.1. Feature Analysis

A total of 44 features were collected.  Table 1 lists the mean values, median values, and standard deviations of the features concerning the low-grade and high-grade tumor lesions. Regarding the textural features, the numbers in the parentheses denote the associated displacement vectors. Student's *t*-test and the Mann-Whitney *U* test were applied to evaluate the differences in feature values between the tumors of different grades. The tests indicated that the *E*_*R*_ values differed significantly between the low-grade and the high-grade tumors (*P* < 0.05). The *P* values of some features were marginal (e.g., *T*_Cor(1, 1, 1)_, *T*_Cor(0, 0, 1)_, *E*_*RI*_, *P*_*R*std_, and *P*_*Sm*_).

### 3.2. Selected Features

Fourteen features were selected using the proposed GA-based approach: *T*_*A*(1,1,1)_, *T*_Con(1, 1, 1)_, *T*_*I*(1,1,1)_, *T*_Cor(1, 1, 1)_, *T*_Cor(1, 0, 0)_, *T*_*A*(0,1,0)_, *M*_*V*_, *M*_Cd_, *M*_*Rm*_, *E*_*S*_, *E*_*R*_, *E*_*ROa*_, *P*_*Sm*_, and *P*_*Rm*_. The selected feature set contained 6 of the 24 textural features, 3 of the 6 morphological features, 3 of the 9 ellipsoid fitting features, and 2 of the 5 posterior acoustic features, indicating that an appropriate combination of feature types might improve the performance of the CAD system.

### 3.3. Model Performance Evaluation

SVM models were developed using the selected features, all of the available features, all of the morphological features, all of the ellipsoid fitting features, all of the textural features, or all of the posterior acoustic features, separately. During the model development process, the GA was implemented to optimize the model parameters.  Table 2 shows the CV classification performance results of the 6 models. The model that was developed using the selected features outperformed the other models. Practically, high-grade tumors are more severe. Misdiagnosing a high-grade tumor as a low-grade tumor may increase the risk of harm and should be avoided. Therefore, the sensitivity and the NPV are 2 critical indices for evaluating the performance of CAD systems. The model that was developed using the selected features attained reasonable sensitivity (79.31%) and a high NPV (94.50%). The model developed using all of the features was inferior to the model developed using the selected features, possibly because of overfitting (including too many trivial explanatory variables in the model).

## 4. Conclusion

This study proposed a CAD system for discriminating the tumor grades of breast cancer in US images. The effectiveness of the proposed system was verified based on clinical data. The textural, morphological, ellipsoid fitting, and posterior acoustic features of the tumors were quantified using the US images. An SVM classifier was developed using a GA to facilitate feature selection and model parameter optimization. An optimal set comprising 14 features (out of 44 total features) was determined. The proposed CAD system effectively distinguished between high-grade and low-grade tumors at an accuracy of 85.14% (126/148), a sensitivity of 79.31% (23/29), a specificity of 86.55% (103/119), and an *A*_*Z*_ of 0.7940. Additional features, such as the angle of the long axis of the fitted ellipsoid or the abrupt interface between tumor and normal tissue, can be included in future research to further improve the CAD system.

## Figures and Tables

**Table 1:** Values of the features of low- and high-grade tumors.

Features	Low-grade		High-grade		*P* value
	Mean ± SD	Median	Mean ± SD	Median	
Textural					
*T*_*A*(1,1,1)_		0.025		0.026	0.201
*T*_Con(1,1,1)_	3.227 ± 0.696		3.260 ± 0.686		0.815
*T*_*I*(1,1,1)_		0.482		0.485	0.927
*T*_*E*(1,1,1)_	3.936 ± 0.206		3.911 ± 0.149		0.542
*T*_*D*(1,1,1)_	1.360 ± 0.170		1.372 ± 0.147		0.715
*T*_Cor(1,1,1)_	0.623 ± 0.088		0.590 ± 0.078		0.069
*T*_*A*(1,0,0)_		0.031		0.033	0.218
*T*_Con(1,0,0)_	1.874 ± 0.433		1.864 ± 0.354		0.914
*T*_*I*(1,0,0)_	0.583 ± 0.043		0.580 ± 0.032		0.747
*T*_*E*(1,0,0)_	3.727 ± 0.203		3.706 ± 0.151		0.608
*T*_*D*(1,0,0)_	1.002 ± 0.138		1.005 ± 0.108		0.900
*T*_Cor(1,0,0)_	0.783 ± 0.057		0.770 ± 0.041		0.233
*T*_A(0,1,0)_		0.026		0.027	0.214
*T*_Con(0,1,0)_	2.925 ± 0.631		2.959 ± 0.549		0.795
*T*_*I*(0,1,0)_	0.507 ± 0.044		0.501 ± 0.026		0.504
*T*_*E*(0,1,0)_	3.905 ± 0.206		3.887 ± 0.146		0.647
*T*_*D*(0,1,0)_	1.291 ± 0.163		1.305 ± 0.119		0.658
*T*_Cor(0,1,0)_	0.661 ± 0.082		0.631 ± 0.060		0.072
*T*_*A*(0,0,1)_		0.040		0.043	0.496
*T*_Con(0,0,1)_	1.104 ± 0.271		1.134 ± 0.220		0.583
*T*_*I*(0,0,1)_	0.669 ± 0.039		0.661 ± 0.029		0.331
*T*_*E*(0,0,1)_	3.493 ± 0.198		3.489 ± 0.144		0.916
*T*_*D*(0,0,1)_	0.736 ± 0.109		0.754 ± 0.084		0.413
*T*_Cor(0,0,1)_	0.873 ± 0.034		0.860 ± 0.030		0.061
Morphological					
*M*_*V*_		2.596 × 10^3^		2.904 × 10^3^	0.824
*M*_*A*_		1.268 × 10^3^		1.419 × 10^3^	0.783
*M*_*Rm*_	9.259 ± 2.919		9.691 ± 3.774		0.502
*M*_*R*std_		1.533		1.896	0.599
*M*_Cc_	0.377 ± 0.101		0.373 ± 0.111		0.868
*M*_Cd_	0.998 ± 0.002		0.998 ± 0.003		0.111
Ellipsoid fitting					
*E*_*A*_		1.622		1.757	0.521
*E*_*S*_	1.238 ± 0.089		1.225 ± 0.114		0.503
*E*_*V*_	0.910 ± 0.015		0.913 ± 0.018		0.427
*E*_*RO*_		15		18	0.082
*E*_*RI*_		7		10	0.055
*E*_*R*_		23		30	0.047
*E*_*ROa*_		4		4	0.642
*E*_*RIa*_		1		1	0.842
*E*_*Ra*_		5		5	0.595
Posterior acoustic					
*P*_std_	33.459 ± 6.517		34.870 ± 7.021		0.305
*P*_*R*std_	1.438 ± 0.318		1.570 ± 0.408		0.061
*P*_*S*std_	9.841 ± 6.939		12.173 ± 8.391		0.122
*P*_*Sm*_	32.666 ± 26.935		42.986 ± 34.495		0.083
*P*_*Rm*_	1.802 ± 0.780		2.035 ± 0.851		0.158

The mean value, standard deviation (SD), median value, and *P* value of *t*-test or Mann-Whitney U test of each feature. Student's *t*-test was applied if a feature is normally distributed; otherwise, Mann-Whitney *U* test was applied. The Kolmogorov-Smirnov test was applied to normality test.

**Table 2:** Performance of the proposed CAD system when different feature sets were used.

	Feature type					
	Selected	All	Morphological	Ellipsoid fitting	Textural	Posterior acoustic
Accuracy	85.14%	77.03%	66.89%	70.95%	66.22%	78.38%
Sensitivity	79.31%	62.07%	37.93%	41.38%	72.41%	48.28%
Specificity	86.55%	80.67%	73.95%	78.15%	64.71%	85.71%
PPV	58.97%	43.90%	26.19%	31.58%	33.33%	45.16%
NPV	94.50%	89.72%	83.02%	84.55%	90.59%	87.18%
Az	0.7940	0.6953	0.4490	0.5575	0.7068	0.6647

Accuracy = (TP + TN)/(TP + TN + FP + FN); sensitivity = TP/(TP + FN); specificity = TN/(TN + FP); PPV = TP/(TP + FP); NPV = TN/(TN + FN), where TP is true positive (the number of high-grade tumors classified correctly); FN is false negative (the number of high-grade tumors classified incorrectly); FP is false positive (the number of low-grade tumors classified incorrectly); TN is true negative (the number of low-grade tumors classified correctly).
